# Woody Bamboos (Poaceae, Bambusoideae, Bambuseae) Native to Southern South America: A Synopsis

**DOI:** 10.3390/plants15071085

**Published:** 2026-04-01

**Authors:** Andrea Susana Vega, Carolina Guerreiro, José Vicente González Figueroa, Zulma Esther Rúgolo

**Affiliations:** 1 Cátedra de Botánica General, Departamento de Recursos Naturales y Ambiente, Facultad de Agronomía, Universidad de Buenos Aires, Av. San Martín 4453, Buenos Aires C1417DSE, Argentina; jvgfigueroa@agro.uba.ar; 2 Consejo Nacional de Investigaciones Científicas y Técnicas (CONICET), Buenos Aires C1040AAH, Argentina; zrugolo@darwin.edu.ar; 3 Instituto de Botánica Darwinion (CONICET-ANCEFyN), Labardén 200, San Isidro B1642HYD, Argentina

**Keywords:** Argentina, Bolivia, Brazil, Chile, native woody bamboos, Paraguay, taxonomy, Uruguay

## Abstract

Constituting the first synopsis on woody bamboos in southern South America, this contribution provides a comprehensive overview of its 12 native genera. The synopsis includes botanical descriptions for each genus, a complete checklist of the taxa, as well as information on their habitat, uses and applications, and flowering period. Furthermore, it provides keys that allow for the identification of the genera: one based on vegetative characters, and the other on both vegetative and reproductive characters.

## 1. Introduction

Bamboos are one of the single most important groups of plants to the largest number of people in the world. Bamboos have found their way into folklore, rituals, sports, construction, the arts and music, the kitchen and the garden, meeting economic, ecological and spiritual needs [1,2,3,4,5].

Within Poaceae, the subfamily Bambusoideae is composed of three tribes: Arundinarieae (temperate woody bamboos), Bambuseae (tropical woody bamboos), and Olyreae (herbaceous bamboos). The tribe Bambuseae consists of two major clades: the Paleotropical (Old World or Asian) woody bamboos and the Neotropical (New World or American) woody bamboos [6]. The Neotropical woody bamboo clade is further subdivided into the subtribes Arthrostyliidinae, Guaduinae, and Chusqueinae. The Bamboo Phylogeny Group [7], in the classification of bamboo tribes and subtribes, recognized 172 species in 14 genera of Arthrostylidiinae, 160 species in 1 genus of Chusqueinae, and 45 species in 5 genera of Guaduinae. Later, the clasification was updated to include 195 species in 16 genera of Arthrostylidiinae, 193 species in 1 genus of Chusqueinae, and 60 species in 6 genera of Guaduinae [8]. Since then, new genera and species have been described thanks to the advancement of botanical exploration and studies. Presently, the subtribe Arthrostyliidinae is composed of 19 genera (*Actinocladum* McClure ex Soderstr., *Alvimia* C. E. Calderón ex Soderstr. & Londoño, *Arthrostylidium* Rupr., *Athroostachys* Benth., *Atractantha* McClure, *Aulonemia* Goudot, *Aulonemiella* L. G. Clark, Londoño, C. D. Tyrrell & Judz., *Cambajuva* P. L. Viana, L. G. Clark & Filg., *Colanthelia* McClure & E. W. Sm., *Didymogonyx* (L. G. Clark & Londoño) C. D. Tyrrell, L. G. Clark & Londoño, *Elytrostachys* McClure, *Filgueirasia* Guala, *Glaziophyton* Franch., *Merostachys* Spreng., *Myriocladus* Swallen, *Quixiume* C. D. Tyrrell, L. G. Clark, P. L. Viana & Santos-Gonç., *Rhipidocladum* McClure, *Stelanemia* C. D. Tyrrell, L. G. Clark, P. L. Viana & Santos-Gonç., and *Vianaea* C. D. Tyrrell, L. G. Clark, Santos-Gonç. & Afonso). The subtribe Guaduinae comprises six genera (*Apoclada* McClure, *Eremocaulon* Soderstr. & Londoño, *Guadua* Kunth, *Olmeca* Soderstr., *Otatea* (McClure & E. W. Sm.) C. E. Calderón & Soderstr., and *Tibisia* C. D. Tyrrell, Londoño & L. G. Clark), whereas the Chusqueinae includes only a single genus (*Chusquea* Kunth). These perennial genera are distributed from Mexico along Central America and the Caribbean Islands to South America [6,9,10]. They span from northern South America to the southern regions of Chile and Argentina. Within the Western hemisphere, South America has the highest diversity and number of woody bamboo species, which occur in a broad range of environments. Woody bamboos occupy extensive areas of humid, lowland tropical evergreen forests in the Amazonian basin and the Atlantic forests and are understory dominants in several different types of forest throughout South America. They are also characteristic of tropical and subtropical montane forests along the Andes Mountain range, extending southward into the cold temperate beech forests. Bamboo is also represented in drier habitats ranging from seasonally deciduous forests to open grasslands, such as the Brazilian cerrado. Above the tree line, woody bamboos are also the main component of the unique Andean vegetation formations known as páramos [1,9].

Since the publication of the most recent treatment on the diversity, distribution, and classification of Neotropical woody bamboos [11], new findings have been made in the fast-developing area of taxonomy. South America is one of the major global centers of woody bamboo diversity, yet the native woody bamboos of southern South America have not previously been synthesized. Furthermore, an updated identification key for the genera occurring in southern South America is currently lacking. Additionally, bamboo resources in relation to sustainable development and emerging bamboo-based material applications are underexplored, including their relevance to the “bamboo instead of plastic” initiative [12]. For this reason, a review including morphological descriptions, species lists, habitat information, flowering records, uses, distributional data, and identification keys for the woody bamboo genera occurring in this region constitutes the first step to fill an important gap in the literature. Consequently, this review serves as a useful reference for researchers in bamboo systematics, floristics, biodiversity conservation, as well as a crucial foundation for future studies in taxonomy, biogeography, phylogeny, and the evaluation of bamboo resources for multiple purposes.

**Figure 1:**
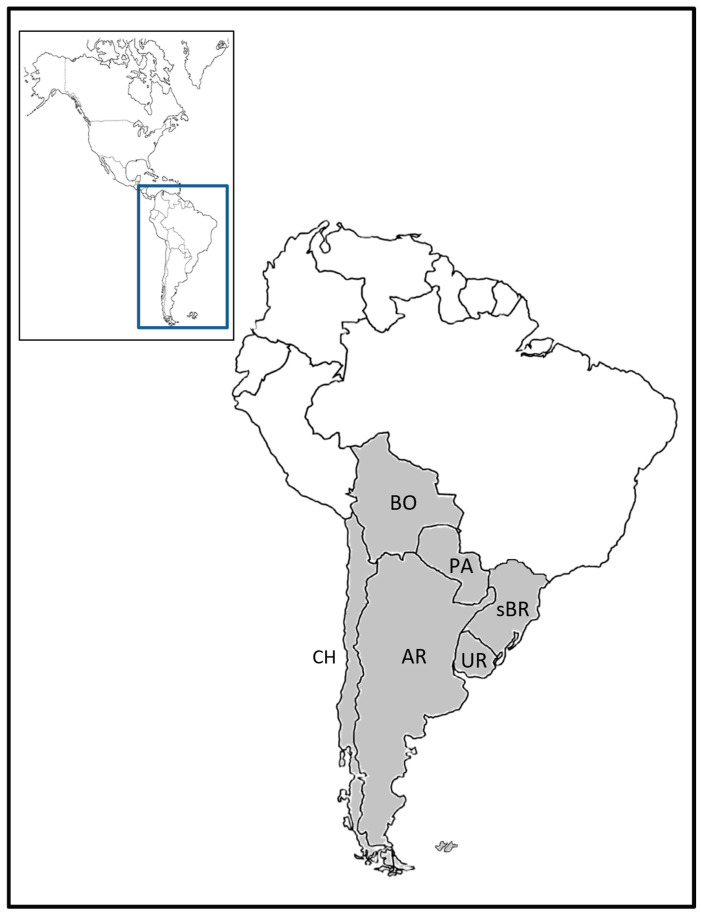
Map showing the study area of native woody bamboos across southern South America. Country codes: AR, Argentina; BO, Bolivia; CH, Chile; PA, Paraguay; sBR, southern Brazil; UR, Uruguay.

**Figure 2:**
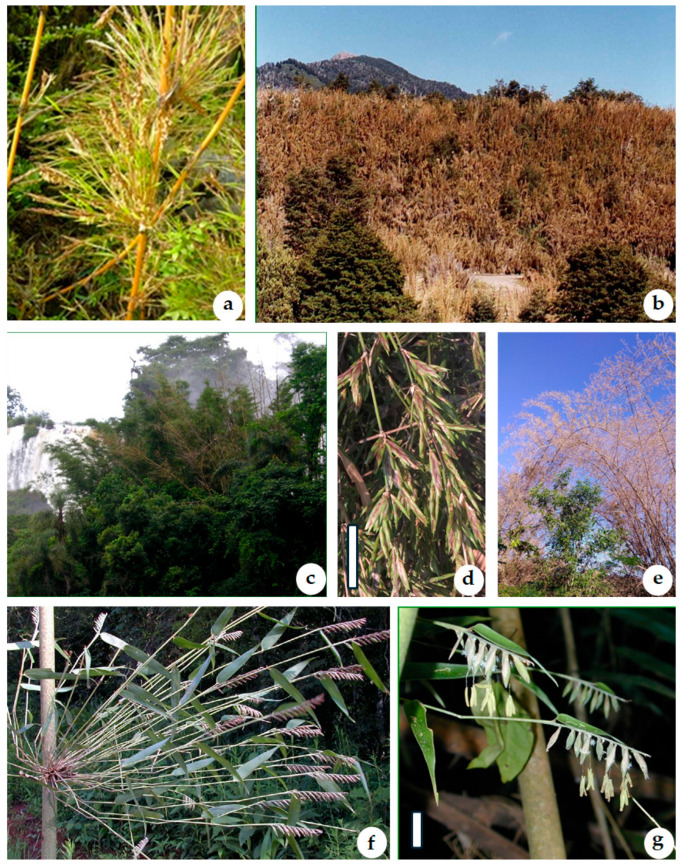
Flowering events in species of Chusqueinae, Guaduinae, and Arthrostylidinae: (**a**,**b**) *Chusquea culeou*; (**a**) flowering branch complement; (**b**) dead clumps after flowering; (**c**) *Guadua chacoensis*; (**d**,**e**) *Guadua trinii*; (**d**) pseudospikelets, detail; (**e**) dead clumps after flowering; (**f**) flowering branch complement in *Merostachys multiramea*; (**g**) spikelets of *Merostachys clausenii* in anthesis. Bars: (**d**) 1 cm; (**g**) 2 cm.

**Figure 3:**
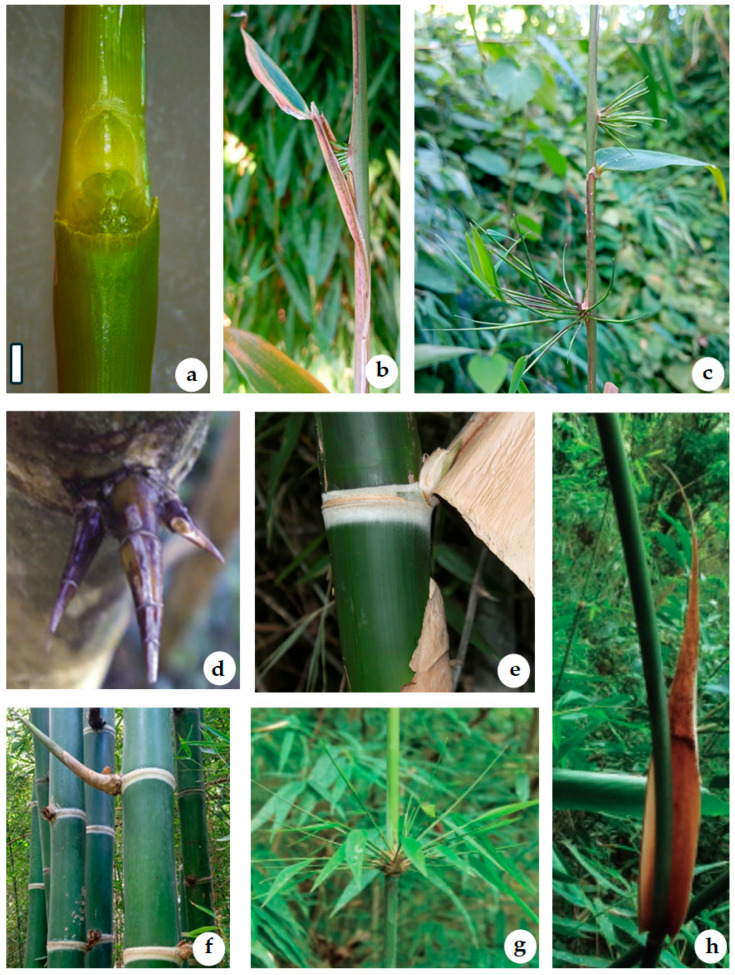
Some diagnostic vegetative characters in species of Chusqueinae, Guaduinae, and Arthrostylidinae: (**a**,**c**) *Chusquea ramosissima*; (**a**) bud complement; (**b**) early developing bud complement; (**c**) early development of the branch complement; (**d**) thorny branch complement in *Guadua variegata*; (**e**,**f**) *Guadua chacoensis*; (**e**) mid-culm node with a stripe of white hairs above and beneath, a solitary axillary bud, and a deciduous culm leaf; (**f**) mid-culm node with an axillary branch; (**g**,**h**) *Rhipidocladum neumannii*; (**g**) branch complement; (**h**) culm leaf. Bar: 1 mm.

Considering that South America has the highest diversity and number of woody bamboo species, the present review provides a comprehensive synopsis of the 12 genera occurring in southern South America (Figure 1). The synopsis includes botanical descriptions for each genus, a complete checklist of the taxa, as well as information on their habitat, uses and applications, and flowering period (Figure 2). The study includes two identification keys: one based on vegetative characters, and the other based on both vegetative and reproductive characters. Some of the diagnostic vegetative characters used in the keys for identification of the genera are illustrated in Figure 3.

## 2. Synopsis

1. **Actinocladum** McClure ex Soderstr.
  **Plants:** 3–4.6 m tall, medium-sized, erect, densely caespitose. **Rhizomes:** pachymorph. **Culms:** hollow, often with a pithy center. **Culm leaves:** sheaths fimbriate at the margin, with fused bases; blades spreading to reflexed, deciduous. **Mid-culm branch complement:** fan-shaped, branches equal in length. **Foliage leaves:** sheaths fimbriate at the margin, with fused bases; blades linear-lanceolate to broad-lanceolate, stiff to deflexed. **Synflorescences:** paniculate or racemose, composed of 2-4(-8) spikelets. **Spikelets:** 6–7.5 cm long, stramineous with light green mottling, narrow, containing 7–10 fertile florets, the uppermost floret rudimentary, long-pedicellate; pedicels up to 4 cm long; glumes short, unequal, ovate, acuminate, many-nerved; lemma ovate-lanceolate, acuminate, many-nerved; palea ovate, acute. **Fruit:** an achenelike caryopsis.
  *Actinocladum* is a monotypic genus [13].
  Species: **Actinocladum verticillatum** (Nees) McClure ex Soderstr. [BO].
  Habitat: The genus is restricted to seasonally dry cerrado vegetation on the Brazilian *planalto* and adjacent Bolivia, in lowlands from 250 to 1250 m [1]. It grows on rocky hillsides or in open stretches, forming dense clumps subjected to long dry seasons and periodic fires [14].
  Uses and applications: Constructions, baskets, kitchen utensils, shoots, medicinal use. Also, culms of *A. verticillatum* are used in the manufacture of arrows in Brazil [14]. The perennial foliage serves as a high-quality forage resource, providing livestock with protein-rich leaves and tender young culm tips [15].
  Flowering period: The estimated flowering cycle is ca. 32 years [16].
2. **Apoclada** McClure.
  **Plants:** 5–8 m tall, small to medium-sized, caespitose. **Rhizomes:** pachymorph. **Culms:** erect. Internodes terete, thick-walled, distally pubescent. Nodes glabrous. **Culm leaves:** sheaths persistent, antrorsely scabrous, without auricles; blades triangular, acuminate, erect, glabrous. **Mid-culm branch complement:** with 3 branches dominant. **Foliage leaves:** sheaths glabrous; blades linear, pubescent. **Synflorescence:** comprising only 1(-2) spikelets; each fertile spikelet subtended by a subtending leaf. **Spikelets:** 2–3 cm long, lanceolate or oblong, comprising 8–12 fertile florets, with a rudimentary floret toward the apex, long-pedicelled; rhachilla internodes pubescent; glumes both absent; fertile lemma ovate, chartaceous, keeled, 9–11-nerved; lemma acuminate, puberulous, hairy on nerves; palea lanceolate, keels ciliolate. **Fruit:** a caryopsis.
  This genus is monotypic [13].
  Species: **Apoclada simplex** McClure & L.B. Sm. [BR].
  Habitat: *Apoclada simplex* grows in sunny places in lower-montane deciduous forests and Paraná pine forests in southern Brazil [1].
  Uses and applications: Arts and crafts. It is also a source of forage [17].
  Flowering period: no information found.
3. **Arthrostylidium** Rupr.
  **Plants:** 0.30–15 m tall, small to medium sized, forming clumps, clambering or scandent. **Rhizomes:** pachymorph with short necks. **Culms:** cylindrical, hollow. **Culm leaves:** sheaths with fimbriae usually not prominent; blades erect and persistent. **Mid-culm branch complement:** with 3–15 branches per node, all arising from a single bud. **Foliage leaves:** sheaths with fimbriae; blades linear-lanceolate or ovate to elliptical, symmetric. **Synflorescences:** spicate or racemose, main axis straight or zig-zagged. **Spikelets:** containing 2–15 fertile florets, subsessile; glumes 1–3, apiculate or awned, glabrous; lemma and palea subequal, lanceolate to ovate, acute. **Fruit:** a caryopsis.
  The genus comprises 29 species [11].
  Species: **Arthrostylidium canaliculatum** Renvoize [BO], **A. ecuadorense** Judz. & L.G. Clark [BO], and **A. venezuelae** (Steud.) McClure [BO].
  Habitat: They grow in a variety of forested habitats from sea level to cloud and elfin forests at elevations of 3700 m [1].
  Uses and applications: *Arthrostylidium venezuelae* and *A. canaliculatum* have applications in basketry and traditional medicine [1,18].
  Flowering period: no information found.
4. **Athroostachys** Benth.
  **Plants:** 3–8 m tall, clambering or scandent. **Rhizomes:** pachymorph. **Culms:** nearly solid, with a very small lumen. **Culm leaves:** sheaths with a darkened or thickened girdle, persistent or tardily deciduous, lacking auricles, with fimbriae present and oral setae absent; blades deciduous and reflexed. **Mid-culm branch complement:** consisting of three branches per node, arranged with a central branch usually slightly larger than the lateral ones, arising from a single bud, promontory absent. **Foliage leaves:** sheaths with the overlapping margin ciliate, summit extensions absent, fimbriae slightly sinuous or radiating in all directions, oral setae absent; blades not tessellate, lanceolate-oblong, symmetrically wide on both sides of the midrib, with a conspicuous green marginal stripe on the abaxial surface, margins antrorsely scabrous. **Synflorescences:** short-pedunculate, contracted-paniculate or capitate. **Spikelets:** subsessile, composed of two glumes, a single fertile floret, and an apical rudimentary floret; glumes subequal, ovate, with acute apices; sterile lemmas absent; fertile floret one, with an elongate rachilla extension bearing a small rudimentary floret; lemma ovate, 7-nerved, pubescent, apex acuminate, awnless; palea 2-nerved, keels ciliate. **Fruit:** not seen.
  The genus comprises 2 species [19].
  Species: **Athroostachys capitata** (Hook.) Benth. [BR].
  Habitat: The species grows in forested environments at low elevations, approximately between 30 and 270 m [20]. It is endemic to Brazil and occurs in the Atlantic Forest, with records documenting its presence near Jacareí, in the state of Paraná [21].
  Uses and applications: no information found.
  Flowering period: no information found.
5. **Aulonemia** Goudot.
  **Plants:** small to large sized, erect or clambering. **Rhizomes:** pachymorph. **Culms:** hollow or solid, all subequally elongated or 1 long internode alternating with 2–4 very short internodes; nodes bearing a single bud. **Culm leaves:** sheaths with long fimbriae; blades triangular, reflexed. **Foliage leaves:** sheaths with fimbriae short to long; blades linear-lanceolate or ovate, ascending to reflexed. **Synflorescences:** paniculate, contracted to open, ovate or pyramidal. **Spikelets:** long-pedicellate; glumes 2–3, ovate to ovate-lanceolate, obtuse to acute to mucronate to awned, unequal; lemmas lanceolate, broadly lanceolate, lanceolate-ovate or ovate-elliptical, usually awned. **Fruit:** a caryopsis.
  This genus comprises 49 spp. [19]. In Austral America, it is distributed in BO and BR.
  Species: **Aulonemia amplissima** (Nees) McClure [BR], **A. aristulata** (Döll) McClure [BR], **A. boliviana** Renvoize [BO], **A. bromoides** Judz. & Shea [BO], **A. cincta** P. L. Viana & Filg. [BR], **A. cochabambensis** Judz. & L. G. Clark [BO], **A. fuentesii** Judz. & Geisth [BO], **A. herzogiana** (Henrard) McClure [BO], **A. insignis** Judz. & L.D. Gibbons [BO], **A. insolita** I. Jiménez, Reátegui & Ruiz-Sanchez [BO], **A. lanciflora** McClure & L. B. Sm. [BR], **A. longipedicellata** Renvoize [BO], **A. madidiensis** Judz., D. C. Ziegler & Zueger [BO], **A. scripta** Judz. & Wayda [BO], and **A. tremula** Renvoize [BO].
  Habitat: *Aulonemia* occurs in wet, montane forests and páramos, from sea level to 3600 m [1].
  Uses and applications: In the Andes, indigenous communities utilize the culms of *A. queko* for crafting musical instruments and the foliage as fodder for livestock [1].
  Flowering period: no information found.
6. **Cambajuva** P. L. Viana, L. G. Clark & Filg.
  **Plants:** 0.2–2.5 m tall, erect. **Rhizome:** pachymorph. **Culms:** hollow, terete, not mottled, smooth to striate, walls thick to thin, glabrous, sometimes covered by a whitish wax, green to glaucous, becoming brown to purplish with age, nodal line horizontal, glabrous; supranodal ridge inconspicuous; bud single per node, triangular to broadly ovate. **Culm leaves:** clearly differentiated from foliage leaves, tardily deciduous; sheaths glabrous, margins glabrous to sparsely ciliate, apex slightly asymmetric; auricles absent; fimbriae erect, straight to slightly undulate, terete to weakly flattened, free toward the base; blades sessile, erect, triangular to ovate-lanceolate. Mid-culm branch complement arising from a single bud, usually borne on a promontory, consisting of 1–7 branches. **Foliage leaves:** sheaths puberulous, ciliate along one margin, keeled toward the apex; summit extensions absent; fimbriae persistent to tardily deciduous, erect, rarely spreading; blades lanceolate, coriaceous, glabrous, glaucous on both surfaces except for a conspicuous green marginal stripe on the abaxial surface, base symmetric to slightly asymmetric, rounded, apex acuminate and pungent, margins smooth to antrorsely scabrous. **Synflorescences:** paniculate, spiciform, contracted, with branches appressed to the main axis. **Spikelets:** ellipsoid, consisting of two glumes, one basal sterile floret, represented by a glumiform lemma and a rudimentary palea, 2–4 fertile florets, and one apical rudimentary floret; glumes and lemmas awned. **Fruit:** not seen.
  This genus is monotypic [19].
  Species: **Cambajuva ulei** (Hack.) P. L. Viana, L. G. Clark & Filg. [BR].
  Habitat: Endemic to southern Brazil, known from the Serra Geral region in the states of Santa Catarina and Rio Grande do Sul. The species occurs primarily in peaty bogs dominated by *Sphagnum* L. and *Polytrichum* Hedw., and more rarely in riparian vegetation.
  Uses and applications: no information found.
  Flowering period: no information found.
7. **Chusquea** Kunth.
  **Plants:** isolated or forming caespitose clumps. **Rhizomes:** pachymorph, leptomorph, or both types in the same species. **Culms:** erect, pendulous at the apex, or leaning, solid, terete (cylindrical in cross-section), grooved, or flattened in the internodes; sometimes mature culms appear to be hollow due to the tearing of the pith; mid-culm nodes with a dominant, larger central bud surrounded by smaller buds, developing numerous extravaginal primary branches. **Culm leaves:** persistent or deciduous; sheath usually papery, without auricles; blade sessile, more or less developed. **Foliage leaves**: sheath usually persistent, summit extensions present or absent, sometimes with small tufts of hairs at the summit; blade lanceolate, acuminate, membranaceous or coriaceous, apex rigid or sharp, margin slightly revolute, smooth or scabrous, central nerve visible or not, as are the transverse veinlets, generally deciduous, petiolate. **Synflorescence:** terminal, paniculate, racemose, or capitate, usually without subtending bracts. **Spikelets:** briefly pedicellate, with four glumes and a single (exceptionally 2), terminal, fertile floret lacking a rachilla extension. Glumes I and II acute, acuminate, aristulate, or obtuse, of variable length, sometimes scale-like or enerved or almost absent; glumes III and IV very similar to each other, acuminate or aristulate; fertile lemma 7–9-nerved, acute, acuminate, and aristulate; palea generally 2–4-nerved, longer or slightly shorter than its lemma, with the back flat or rounded, sulcate toward the apex, which may be bidentate or bimucronate, exceptionally keeled, not sulcate, and with an entire apex. **Fruit:** a caryopsis.
  *Chusquea* comprises 208 spp. [13,22].
  Taxa: **Chusquea andina** Phil. [AR, CH], **C. anelythra** Nees [BR], **C. anelytroides** Rupr. ex Döll [BR], **C. argentina** Parodi [AR, CH], **C. asymmetrica** (L.G. Clark) L.G. Clark [BO], **C. bambusoides** (Raddi) Hack. [BR], **C. bicentenaria** I. Jiménez & M. Zárate [BO], **C. capitata** Nees [BR], **C. capituliflora** Trin. [BR], **C. ciliata** Phil. [CH], **C. culeou** E. Desv. [AR, CH], **C. cumingii** Nees [CH], **C. deficiens** Parodi [AR, BO], **C. delicatula** Hitchc. [BO], **C. depauperata** Pilg. [BO], **C. egluma** Guerreiro & Rúgolo [AR], **C. fernandeziana** Phil. [CH], **C. floribunda** Guerreiro & Rúgolo [AR, BO], **C. gigantea** Demoly [CH], **C. gracilis** McClure & L. B. Sm. [BR], **C. hatschbachii** L. G. Clark [BR], **C. hystrix** L. G. Clark, McMurchie & Baya [BO], **C. ibiramae** McClure & L. B. Sm. [BR], **C. juergensii** Hack. [AR, BR, UR], **C. kleinii** A. C. Mota, R. P. Oliveira & L. G. Clark [BR], **C. laegaardii** (L. G. Clark) L. G. Clark [BO], **C. leptophylla** Nees [BR], **C. longipendula** Kuntze [BO], **C. lorentziana** Griseb. [AR, BO], **C. macrostachya** Phil. [CH], **C. meyeriana** Rupr. ex Döll [BR], **C. mimosa** McClure & L. B. Sm. subsp. **mimosa** [BR], **C. mimosa** subsp. **australis** L. G. Clark [BR], **C. montana** Phil. f. **montana** [AR, CH], **C. montana** Phil. f. **nigricans** (Phil.) Matthei [CH], **C. nudiramea** L. G. Clark [BR], **C. oligophylla** Rupr. [BR], **C. ovatifolia** Attigala, A. Fuentes & L.G. Clark [BO], **C. oxylepis** (Hack.) Ekman [BR], **C. parodii** A. S. Vega & Rúgolo [BO], **C. paucispiculata** A. S. Vega & Rúgolo [BO], **C. peruviana** E.G. Camus (BO], **C. picta** Pilg. [BO], **C. pinifolia** (Nees) Nees [BR], **C. quila** Kunth [AR, CH], **C. ramosissima** Lindm. [AR, BR, PA, UR], **C. renvoizei** L.G. Clark [BO], **C. scandens** Kunth [BO], **C. sellowii** Rupr. [BR], **C. serrulata** Pilg. [BO], **C. spicata** Munro [BO], **C. tenella** Nees [AR, BR, UR], **C. tenuiglumis** Döll [BR], **C. tessellata** Munro [BO], **C. uliginosa** Phil. [CH], **C. uniflora** Steud. [AR, BO], **C. urelytra** Hack. [BR], **C. valdiviensis** E. Desv. [AR, CH], **C. windischii** L. G. Clark [BR], and **C. yungasensis** L. G. Clark & A. C. Mota [BO].
  Habitat: Its distribution ranges from Mexico to Argentina and Chile, representing the widest latitudinal range among bamboo genera in the Americas (24° N to 47° S). Some species extend to the southernmost part of South America within the Andean-Patagonian forests of Argentina (as far south as the Río Negro province) and Chile (down to the XI Aysén Region). In these areas, they are associated with *Nothofagus* Blume forests or their peripheries, acting as endemic species to the region and the dominant component of the understory [23]. In Uruguay, three species are distributed as far as south as Maldonado department. *Chusquea* is primarily montane, including cloud and elfin forests. Some species grow in humid páramos and campos de altitude, while others are found in lowland tropical habitats and temperate forests at higher latitudes.
  Uses and applications: Floors, tables, armchairs, wall covering, gardening, bakestry.
  Flowering period: The available records show different life cycle estimates in several species of *Chusquea*, such as ca. 15 years (in *C. capituliflora*, *C. tenella*), ca. 30 years (*C. lorentziana*, *C. meyeriana*, *C. ramosissima*), ca. 45 years (*C. montana*, *C. quila*) and ca. 60 years (*C. argentina*, *C. culeou*, *C. valdiviensis*) [16] (Figure 2a,b).
8. **Colanthelia** McClure & E. W. Sm.
  **Plants:** 0.5–10 m tall, perennial. **Rhizomes:** pachymorph. **Culms:** hollow, erect or scandent, smooth; nodes prominent with a narrow rim, mid-culm nodes with a single initial bud that produces a dominant axis over the secondary axes originating from its proximal buds. **Culm leaves:** mid-culm sheaths persistent; blades reflexed, deciduous. **Foliage leaves:** blades lanceolate, not manifestly tessellate. **Synflorescence:** terminal, racemose or contracted-paniculate, with few to many spikelets. **Spikelets:** pedicellate, pluri- or pauciflowered, narrow, fragile; glumes (1-)2(-3). Lodicules 3, the 2 anterior ones asymmetrical and the posterior one symmetrical, smaller in size; androecium 3; gynoecium with 2 stigmas; **Fruit:** a caryopsis.
  *Colanthelia* is composed of 10 spp. [19] that inhabit the Atlantic rainforest domain, occurring in southern and southeastern Brazil with only one, *C. rhizantha* (Hack.) McClure, occurring, in the province of Misiones (Argentina).
  Species: **Colanthelia cingulata** (McClure & L. B. Sm.) McClure [BR], **C. intermedia** (McClure & L. B. Sm.) McClure [BR], and **C. rhizantha** (Hack.) McClure [AR, BR].
  Habitat: It grows in forests, along the margins of streams [24].
  Uses and applications: no information found.
  Flowering period: no information found.
9. **Guadua** Kunth.
  **Plants:** up to 30 m tall, perennial, forming dense, spiny clumps. **Rhizomes:** pachymorph. **Culms:** up to 22 cm in diameter, with an erect base and a leaning apex, rough or smooth; internodes cylindrical, sometimes grooved above the bud, hollow, occasionally subsolid, exceptionally multifistulose; mid-culm nodes with a line of white hairs below, typically with a solitary axillary bud that gives rise to a dominant shoot, branched or unbranched at its basal nodes. **Culm leaves:** sheaths auriculate or not, with sinuous fimbriae; blade reduced, persistent or deciduous. **Foliage leaves:** sheath auriculate or not, glabrous or pubescent, fimbriate at summit; blade lanceolate, tessellate or not. **Synflorescence:** with pseudospikelets grouped on lateral or terminal leafy or leafless branches, with a basal bract, a prophyll, and several bracts. **Pseudospikelets:** pluriflowered, apical florets reduced or sterile; glumes 0–1; sterile lemmas 0–1, like the fertile ones but smaller; fertile lemma pluri-nerved, embracing the base of the palea at the maturity of the pseudospikelet, apex apiculate, mucronate, or acute; palea 2-keeled, keels winged. **Fruit:** a dry or bacoid caryopsis.
  The genus comprises 36 spp. [25] and ranges from northern Mexico to northern Argentina and Uruguay, with the greatest diversity found in the Amazon region of Colombia, Venezuela, Ecuador, Brazil, Peru, and Bolivia [26]. In Argentina, it reaches its southernmost natural distribution limit in Entre Ríos province, while in Uruguay, it extends as far south as Montevideo.
  Species: **Guadua chacoensis** (Rojas Acosta) Londoño & P. M. Peterson [AR, BO, BR, PA, UR], **G. chaparensis** Londoño & En. Zurita [BO], **G. glomerata** Munro [BO], **G. paniculata** Munro [AR, BO, BR], **G. paraguayana** Döll [AR, BO, BR, PA], **G. refracta** Munro [BO], **G. sarcocarpa** Londoño & P.M. Peterson [BO], **G. superba** Huber [BO], **G. tagoara** (Nees) Kunth [AR, BR], **G. trinii** (Nees) Nees ex Rupr. [AR, BO, BR, UR], **G. variegata** Lizarazu [AR], and **G. weberbaueri** Pilg. [BO].
  Habitat: *Guadua* species in southern South America occur predominantly in humid to subhumid environments, most frequently associated with riparian systems and gallery forests along rivers and streams. They are characteristic elements of lowland to montane forests, including Atlantic Forest formations and interior subtropical forests, where they often form dense, locally dominant clumps. Several species inhabit seasonally flooded areas, river margins, and forest edges, while others extend into the interior of closed forests. In this region, *Guadua* species are recorded from near sea level to approximately 1200 m.
  Uses and applications: Among *Guadua* species, *G. angustifolia* Kunth is considered the most versatile and useful bamboo. Its close relative, *G. chacoensis* appears to have similar economic potential. The culms are used in construction and crafts, as well as in the manufacturing and decoration of houses and in the making of rustic furniture [2,27]. Discarded culms were successfully used for biochar remediation of aqueous As(V) [4]. Additionally, foliage leaf blades were proposed as a promissory forage [3] and are part of the diet of capuchin monkeys [5].
  *Guadua trinii* is used in the construction of “rancho walls” as mud adheres to its spines and roughness [28]; it has also been utilized in the industry for paper manufacturing [17].
  Flowering period: *Guadua chacoensis*, *G. paraguayana*, *G. sarcocarpa*, *G. tagoara*, *G. trinii* and *G. weberbaueri* are known to have life cycles of ca. 30 years [16] (Figure 2c–e).
10. **Merostachys** Spreng.
  **Plants**: erect, pendent at the distal end. **Rhizomes:** pachymorph. **Culms:** clambering, scabrous, subglabrous or pilose; internodes hollow, rarely solid. **Culm leaves:** sheaths deciduous. **Mid-culm branch complement:** flabellate. **Foliage leaves:** sheaths glabrous or subglabrous, distinctly furrowed, ciliate margins ciliate; oral setae persistent, circinate, whitish or reddish. **Synflorescences:** racemose, subfalcate, terminal, with spikelets oriented unilaterally. **Spikelets:** sessile or subsessile, generally uniflowered; glumes 2, typically unequal; lower glume 1-nerved, glabrous or pubescent; upper glume apiculate, pilose, with reddish or whitish hairs; fertile lemma with acuminate or acute apex, clasping the palea at its base, glabrous or pilose, with reddish hairs; palea biaquillate, apex pilose. **Fruit:** a caryopsis.
  *Merostachys* comprises 58 spp. [22] and it is endemic to the tropical regions of the Americas. Most of the species are distributed in South America and only a few in Mexico and Central America [29]. The southernmost boundary of its range extends to northeastern Argentina (Misiones province), where two species occur.
  Species: **Merostachys caucaiana** Send. [BR], **M. ciliata** McClure & L. B. Sm. [BR], **M. claussenii** Munro [AR, BR, PA], **M. fischeriana** Rupr. ex Döll [BR], **M. fistulosa** Döll [BR], **M. glauca** McClure & L. B. Sm. [BR], **M. kleinii** Send. [BR], **M. kunthii** Rupr. [BR], **M. multiramea** Hack. [AR, BR, PA], **M. neesii** Rupr. [BR], **M. nigricans** I. Jiménez & Vinic.-Silva [BO], **M. petiolata** Döll [BR], **M. pilifera** Send. [BR], **M. pluriflora** Munro ex E. G. Camus [BR], **M. sellovii** Munro [PA], **M. skvortzovii** Send. [BR], **M. speciosa** Spreng. [BR], **M. ternata** Nees [BR], **M. vestita** McClure [BR], and **M. yungasensis** Lizarazu [BO].
  Habitat: The species are mainly found in tropical forests with annual precipitation ranging from 1500 to 2000 mm, an average temperature between 16 and 22 °C, and at elevations varying from 95 to 1403 m [2]. Brazil is the largest center of diversity [1].
  Uses and applications: Leaf blades of *M. claussenii* constitute a mediocre forage while its culms are useful to roof houses [30]. Culms are also employed in wall construction and in crafting baskets and sieves. The culms of *M. multiramea* are used for baskets, crafts, and fodder for livestock [17,31].
  Flowering period: *Merostachys claussenii*, *M. multiramea* and *M. skvortzovii* estimated life cycles are 32 years [16] (Figure 2f,g).
11. **Quixiume** C. D. Tyrrell, L. G. Clark, P. L. Viana & Santos-Gonç.
  **Plants:** 1.5–6.5 m tall, erect to weakly clambering, forming dense clumps. **Culms:** hollow, thin-walled, terete, glabrous, papillose, subequally elongated, green to violaceous, sometimes mottled; nodes with a conspicuous supranodal ridge. **Culm leaves:** weakly differentiated or only gradually differentiated from foliage leaves. **Branch complement:** consisting of a single branch per node, occasionally with smaller-diameter secondary branches at basal nodes. **Foliage leaves:** sheaths slightly keeled, glabrous or sometimes pubescent toward the apex; fimbriae basally flattened, scaberulous, free to completely fused; blades lanceolate to linear-lanceolate, spreading to deflexed, chartaceous, distinctly tessellate, glabrous, adaxially green, abaxially glaucous with a conspicuous marginal green stripe. **Synflorescences:** paniculate, oblong, contracted to open, with first-order branches appressed to the main axis. **Spikelets:** 2.5–5.2 cm long, linear, with 4–10 fertile florets and a terminal rudimentary floret; glumes 2–3, awned, glabrous to scaberulous; lemma 0–1. Fertile lemmas 7–9(–11)-nerved, awned, puberulous to glabrescent, sometimes purplish or lead-colored. Paleas 2-nerved, glabrous, with scaberulous keels toward the apex, sometimes exceeding the lemma. **Fruit:** unknown.
  *Quixiume* is endemic to southeastern and southern Brazil, occurring in montane regions of the Atlantic Forest domain. This genus is monotypic [19].
  Species: **Quixiume radiata** (Rupr.) C. D. Tyrrell, L. G. Clark, P. L. Viana & Santos-Gonç. [BR].
  Habitat: It inhabits humid montane forests, primarily dense and mixed ombrophilous forests, as well as open high-elevation environments between 800 and 2000 m.
  Uses and applications: It provides fodder for cattle and tapirs [17].
  Flowering period: Evidence suggests a reproductive cycle of approximately 12 years, with occasional sporadic flowering events [19].
12. **Rhipidocladum** McClure.
  **Plants:** erect in the proximal part, pendent in the distal part. **Rhizome:** pachymorph. Internodes cylindrical, hollow. Nodes simple or double. **Culm leaves:** sheath lacking auricles or fimbriae (except in section Didymogonyx); blade triangular, rigid, erect or reflexed, persistent or deciduous, papery. Mid-culm axillary buds adnate, solitary, giving rise to a simple shoot. **Mid-culm branch complement:** with secondary branchlets having a flabelliform arrangement. **Foliage leaves:** sheaths with an obtuse apex, usually fimbriate, glabrous or pubescent; blades pseudopetiolate, linear, sometimes ovate-lanceolate, glabrous or pubescent, adaxial surface green, the abaxial surface glaucous, not tessellate, margins serrulate or strigose. **Synflorescences:** racemose, with leafy branches; rachis straight, twisted, or sinuous; subtending bracts of the basal spikelets usually present, the distal ones often absent; prophylls sometimes present. **Spikelets:** 2–12 flowered, laterally compressed; glumes 2-3(-5), equal or not, in a transitional series of short or developed bracts, acute or acuminate, muticous or aristate, 3-plurinerved. Lower fertile florets developed, 1–2 apical ones reduced and sterile; fertile lemma papery, plurinerved, obtuse, mucronate or aristate, covering the palea, mainly in the lower third; palea papery, widely furrowed, biaquillate, the margins overlapping or not. **Fruit:** a dry caryopsis.
  In the Americas, the genus is composed of 21 spp. [13,19].
  Species: **Rhipidocladum harmonicum** (Parodi) McClure [BO], **R. neumannii** Sulekic, Rúgolo & L. G. Clark [AR, BO], **R. parviflorum** (Trin.) McClure [BO, BR], and **R. racemiflorum** (Steud.) McClure [AR, BO].
  Habitat: *Rhipidocladum* inhabits forests from sea level up to 2900 m.
  Uses and applications: Culms are used in basketry. Additionally, the pan pipes and quena flutes characteristic of Andean music are made from *Rhipidocladum harmonicum* culms [1].
  Flowering period: *Rhipidocladum neumannii* and *R. parviflorum* flower regularly every ca. 20 years [16].

### 2.1. Key to the Genera of Southern South America Woody Bamboos Based on Vegetative Characters

1 Plants large-sized, erect, sometimes with scandent culms, usually armed with sharp branch thorns. Culms with a band of short white hairs above and below the nodes … *Guadua*

1’ Plants small, medium to large-sized, culms erect or scandent. Culm branches unarmed, without a band of white hairs at the nodes … 2

2 Culms solid. Rhizomes pachymorph or leptomorph, sometimes on the same plant. Mid-culm nodes with a central bud surrounded by several subsidiary buds (bud complement), arranged symmetrically on both sides of the central bud. The central bud develops a branch that predominates over the rest of the branches … *Chusquea*

2’ Culms hollow (solid in some species of *Arthrostylidium*). Rhizomes pachymorph. Mid-culm nodes with a solitary bud that gives rise to a single axillary branch, sometimes accompanied by smaller lateral branches … 3

3 Branch complement at mid-culm nodes consisting of only one branch, exceptionally more than one in some species … *Aulonemia*

3’ Branch complement at mid-culm nodes consisting of a few to many branches … 4

4 Branches of the branch complement slender, all equal or subequal. Mid-culm nodes with a flattened initial bud, adnate to the surface of the culm, which develops secondary branches, giving rise to a mid-culm fan-shaped branch complement … 5

4’ Branches of the branch complement of two sizes, a dominant larger branch and subsidiary ones, several times smaller. Mid-culm nodes with a prominent initial bud which develops secondary branches not arranged in a fan shape … 7

5 Culm and foliage leaf sheaths with fimbriae fused. Culm leaf blades spreading to reflexed. Foliage leaf blades dimorphic, those of the terminal node much larger than those of the lateral branches … *Actinocladum*

5’ Culm and foliage leaf sheaths lacking fimbriae fused. Foliage leaf blades homomorphic, all about the same size … 6

6 Culms often robust and strong-walled, frequently with scabrous and mottled internodes. Culm leaves with blades lanceolate and reflexed, constricted at the base, narrower than the sheath summit, and deciduous … *Merostachys*

6’ Culms usually weak and thin walled, with smooth, generally unmottled internodes. Culm leaves with blades triangular and erect, confluent with the sheath summit and persistent … *Rhipidocladum*

7 Plants with a mid-culm branch complement usually 2 per node, both dominant. Culm leaf blades triangular, apex acuminate, erect, glabrous. Foliage leaf blades linear, pubescent … *Apoclada*

7’ Plants with a mid-culm branch complement of 3 or more branches … 8

8 Plants with a mid-culm branch complement of 3 subequal branches, the central one slightly dominant. Culm leaves and foliage leaves with dense, long oral setae … *Athroostachys*

8’ Plants with a mid-culm branch complement of more than 3 branches, the central one very much larger and dominant over the secondary ones … 9

9 Nodal region delimited by 2 ridges, the upper one more prominent than the lower, forming a narrow crest on the opposite side of the branch complement; nodes with a girdle, inconspicuous in some species; secondary branches crowded at the base and sides of the main branch; blade of culm leaf petiolate, reflexed … *Colanthelia*

9’ Nodal region without a prominent crest and without a girdle. Branch complement with many secondary branches verticillate at the base of the central, dominant axis; blade of culm leaf erect or reflexed … 10

10 Culm leaves well differentiated from foliage leaves. Culm leaf blades erect. Foliage leaf blades tessellate or not, glaucous or not on both surfaces … 11

10’ Culm leaves scarcely differentiated from foliage leaves, only gradually differentiated from foliage leaves. Culm leaf blades reflexed. Foliage leaf blades strongly tessellate, abaxially glaucous with a conspicuous marginal green stripe … *Quixiume*

11 Plants erect. Mid-culm branch complement consisting of 1–7 branches per node. Foliage leaf blades narrowly lanceolate, apex acuminate and pungent, strongly tessellate especially on their abaxial side, glaucous on both surfaces except for a conspicuous green marginal stripe on the abaxial surface … *Cambajuva*

11’ Plants climbing or hanging from trees. Mid-culm branch complement with 3–15 branches per node. Foliage leaf blades linear-lanceolate or ovate to elliptical, inermous, tessellate or not, not glaucous … *Arthrostylidium*

### 2.2. Key to the Genera of Southern South America Woody Bamboos Based on Vegetative and Reproductive Characters

1 Plants large-sized, erect, sometimes with scandent culms, usually armed with sharp branch thorns. Culms with a band of short white hairs above and below the nodes. Synflorescences composed of pseudospikelets, each one with several functional florets. Palea with winged keels … *Guadua*

1’ Plants small, medium to large-sized, culms erect or scandent. Culm branches unarmed, without a band of white hairs at the nodes. Synflorescences composed of spikelets. Palea with unwinged keels or narrowly winged … 2

2 Culms solid. Rhizomes pachymorph or leptomorph, sometimes on the same plant. Mid-culm nodes with a central bud surrounded by several subsidiary buds (bud complement), arranged symmetrically on both sides of the central bud. The central bud develops a branch that predominates over the rest of the branches. Spikelets with four glumes and a single (exceptionally 2) terminal fertile floret lacking a rachilla extension … *Chusquea*

2’ Culms hollow (solid in some species of *Arthrostylidium*). Rhizomes pachymorph. Mid-culm nodes with a solitary bud that gives rise to a single axillary branch, sometimes accompanied by smaller lateral branches. Spikelets with 1–12 fertile florets … 3

3 Branch complement at mid-culm nodes consisting of only one branch, exceptionally more than one in some species. Spikelets 0.8–5 cm long, mottled or solid-colored, long-pedicelled, containing 2–12 fertile florets, rudimentary florets 1 to several. Fruit a caryopsis … *Aulonemia*

3’ Branch complement at mid-culm nodes consisting of a few to many branches … 4

4 Branches of the branch complement slender, all equal or subequal. Mid-culm nodes with a flattened initial bud, adnate to the surface of the culm, which develops secondary branches, giving rise to a mid-culm fan-shaped branch complement … 5

4’ Branches of the branch complement of two sizes, a dominant larger branch and subsidiary ones, several times smaller. Mid-culm nodes with a prominent initial bud which develops secondary branches not arranged in a fan shape … 7

5 Culm and foliage leaf sheaths with fimbriae fused. Culm leaf blades spreading to reflexed. Foliage leaf blades dimorphic, those of the terminal node much larger than those of the lateral branches. Spikelets 6–7.5 cm long, stramineous with light green mottling, long-pedicelled, containing 7–10 florets, the uppermost floret rudimentary. Fruit an achenelike caryopsis … *Actinocladum*

5’ Culm and foliage leaf sheaths lacking fimbriae fused. Foliage leaf blades homomorphic, all about the same size. One-sided paniculate synflorescence (i.e., comblike) … 6

6 Culms often robust and strong-walled, frequently with scabrous and mottled internodes. Culm leaves with blades lanceolate and reflexed, constricted at the base, narrower than the sheath summit, and deciduous. Spikelets provided with 1–2 fertile florets. Fruit an achenelike caryopsis … *Merostachys*

6’ Culms usually weak and thin walled, with smooth, generally unmottled internodes. Culm leaves with blades triangular and erect, confluent with the sheath summit and persistent. Spikelets provided with several fertile florets. Fruit a caryopsis … *Rhipidocladum*

7 Plants with a mid-culm branch complement usually 2 per node, both dominant. Culm leaf blades triangular, apex acuminate, erect, glabrous. Foliage leaf blades linear, pubescent. Spikelets 2–3 cm long, long-pedicelled, containing 8–12 fertile florets, ending in a rudimentary floret. Glumes absent … *Apoclada*

7’ Plants with a mid-culm branch complement of 3 or more branches … 8

8 Plants with a mid-culm branch complement of 3 subequal branches, the central one slightly dominant. Culm leaves and foliage leaves with dense, long oral setae. Synflorescence condensed, capitate. Spikelets 1–2-flowered … *Athroostachys*

8’ Plants with a mid-culm branch complement of more than 3 branches, the central one very much larger and dominant over the secondary ones. Synflorescence spicate, racemose or paniculate … 9

9 Nodal region delimited by 2 ridges, the upper one more prominent than the lower, forming a narrow crest on the opposite side of the branch complement; nodes with a girdle, inconspicuous in some species; secondary branches crowded at the base and sides of the main branch; blade of culm leaf petiolate, reflexed. Synflorescence terminal, racemose or contracted-paniculate, with few to many spikelets. Spikelets pedicellate, pluri- or pauciflowered … *Colanthelia*

9’ Nodal region without a prominent crest and without a girdle. Branch complement with many secondary branches verticillate at the base of the central, dominant axis; blade of culm leaf erect or reflexed … 10

10 Culm leaves well differentiated from foliage leaves. Culm leaf blades erect. Foliage leaf blades tessellate or not, glaucous or not on both surfaces … 11

10 Culm leaves scarcely differentiated from foliage leaves, only gradually differentiated from foliage leaves. Culm leaf blades reflexed. Foliage leaf blades strongly tessellate, abaxially glaucous with a conspicuous marginal green stripe. Spikelets 2.5–5.2 cm long, linear, composed of 4–10 fertile florets and a terminal rudimentary floret … *Quixiume*

11’ Plants erect. Mid-culm branch complement consisting of 1–7 branches per node. Foliage leaf blades narrowly lanceolate, erect, apex acuminate and pungent, strongly tessellate especially on their abaxial side, glaucous on both surfaces except for a conspicuous green marginal stripe on the abaxial surface. Spikelets 1.8–2.2 cm long, ellipsoid, composed of one basal sterile floret, 2–4 fertile florets, and one apical rudimentary floret … *Cambajuva*

11’ Plants climbing or hanging from trees. Mid-culm branch complement with 3–15 branches per node. Foliage leaf blades linear-lanceolate or ovate to elliptical, lax or reflexed, inermous, tessellate or not, not glaucous. Spikelets with 2–15 fertile florets … *Arthrostylidium*

## 3. Materials and Methods

Area of study. This study focuses on the native woody bamboos that inhabit Southern South America (i.e., Austral America). This region includes Argentina (AR), Bolivia (BO) Chile (CH), Paraguay (PA), Uruguay (UR), and the bordering regions of Brazil (BR; States Rio Grande do Sul, Santa Catarina, and Paraná).

Woody bamboos genera. The information provided for each genus includes the correct name for use, a brief botanical description, geographical distribution and habitat, list of species/taxa, uses and applications, and flowering period. Flowering periods for South American woody bamboos remain largely unknown [16]. Existing information is derived from literature reviews and the study of herbarium specimens housed in major institutions in Argentina and worldwide. This knowledge gap reflects a broader lack of documented data. Furthermore, information on bamboo utilization in the Americas is scarce, indicating that this resource remains largely underexplored and underutilized in some countries.

The taxa list for each genus is based on updated taxonomic treatments and Floras, as well as on databases [22,32,33,34].

## Data Availability

No new data were created or analyzed in this study.
